# Physical activity related to mastery and vitality in a Swedish adult population with economic difficulties

**DOI:** 10.1186/s12889-021-12194-6

**Published:** 2021-11-30

**Authors:** Lisbeth M. Johansson, Hans Lingfors, Marie Golsäter, Margareta Kristenson, Eleonor I. Fransson

**Affiliations:** 1 Unit for Research and Development in Primary Care, Futurum - Academy for Health and Care, Region Jönköping County, Jönköping, Sweden; 2 Linköping University, Linköping, Sweden; 3 The A.D.U.L.T. Research Group, School of Health and Welfare, Jönköping University, Box 1026, 551 11 Jönköping, Sweden; 4 Child Research Group, School of Health and Welfare, Jönköping University, Jönköping, Sweden; 5 Futurum - Academy for Health and Care, Region Jönköping County, Jönköping, Sweden; 6 Unit for Health, Medicine and Care, Department of Medicine and Health, Linköping University, Linköping, Sweden; 7 The A.D.U.L.T Research Group, Department of Natural Science and Biomedicine, School of Health and Welfare, Jönköping University, Jönköping, Sweden

**Keywords:** Physical activity, Psychological resources, Mastery, Vitality, Health promotion and salutogenesis

## Abstract

**Background:**

People with low socio-economic status report lower levels of physical activity (PA). There is insufficient knowledge about the availability of psychological resources for those who are physically active despite having a low socio-economic status. The aim of this study is to investigate the association between PA level and mastery and vitality, respectively, within an adult population with self-reported economic difficulties.

**Method:**

Data from a cross-sectional, population-based study (*n* = 817) were used. Linear regression was used to estimate the unstandardised regression coefficient (*β*) with 95% confidence intervals (95% CI), describing associations between PA levels (independent variable) and scale scores of psychological resources in terms of mastery and vitality (outcome variables). Three models were constructed: Model I unadjusted; Model II adjusted for sex and age; and Model III adjusted for sex, age, smoking and food quality.

**Result:**

After adjusting for sex, age, smoking and food quality and using low-level PA as the reference, high-level PA, but not intermediate-level PA, was related to higher scale scores of mastery (*β* = 0.72 [95% CI 0.08 to 1.37]). For vitality, both high-level PA and intermediate-level PA were related to higher scale scores (*β* = 9.30 [95% CI 5.20 to 13.40] and *β* = 6.70 [95% CI 1.40 to 12.00] respectively).

**Conclusion:**

In an adult population with self-reported economic difficulties, higher levels of physical activity were related to higher mastery and vitality. Our results support that the association between physical activity and psychological resources in terms of mastery and vitality should be considered in the context of targeted health dialogues.

**Trial registration number:**

Not applicable.

**Supplementary Information:**

The online version contains supplementary material available at 10.1186/s12889-021-12194-6.

## Introduction

In Europe, lifestyle-related diseases account for two-thirds of all premature mortality in non-communicable diseases (NCD) such as cardiovascular disease, diabetes, cancer and chronic lung disease. These diseases therefore constitute the major burden on European countries’ health systems, economic development and the well-being of many people. Their prevalence is also more common in groups with lower socio-economic status (SES). These diseases also increase the health inequalities in the population, between socio-economic groups and sexes, and between countries in Europe. It has been estimated that at least 80% of the risk of cardiovascular disease and type II diabetes and 40% of all cancers can be explained by health-related behaviours [1].

Health promotion, according to the Ottawa Charter for Health Promotion, which is a ‘process of enabling people to increase control over, and to improve, their health that “goes beyond healthy life-styles to well-being”’. One goal of health promotion work is to reduce inequality in health [2]. Health promotion work takes its starting point in the salutogenic perspective. A salutogenic perspective means to enabling people with their individual resources and aims to strengthen the potential of people to improve and maintain their good health. This process not only includes the perception of control over health but also identifies a person’s resources to handle different needs [3]. Health is described by Antonovsky as a continuum with the poles ease and dis-ease [4]. When personal resources are used, health can be strengthened and a movement towards the healthy pole takes place. One part of this health continuum is action implication, which contains both efforts of reduction of known risk factors and active interventions [4].

Today, interventions to support health-related behavioural changes such as physical activity (PA), food habits, smoking habits and alcohol use are increasingly important in health promotion [1, 5]. As unhealthy behaviours are more prominent in groups with low SES, they are also important to reducing health inequality [6]. One important intervention to support behavioural changes is health-promoting encounters [7]. The synergy between a person’s awareness of risks and his or her psychological resources could start a salutogenic process in which the person’s resources are used to reduce the effects of his or her risk factors [5]. Important factors in these interventions are whether people are confident that these changes will result in health benefits and are willing and motivated to change their health behaviours. If people feel that health is beyond their control, then health promotion work in these terms will tend to be less successful [8].

General resistant resources (GRR), introduced by Antonovsky, can be described as the property of a person, a collective or a situation which, as evidence or logic has indicated, facilitates successful coping with the inherent stressors of human existenceʼ [9]. The economy is an important element of the GRR concept, and economic difficulties could be a factor that reduces GRR [4]. Today, the salutogenic perspective has also taken other resources under its umbrella, such as the possibility of making changes in life to achieve better health [10]. Antonovsky was inspired by coping strategies such as mastery when he developed the term *manageability* and stated that successful coping strategies are a salutogenic strength, and he saw mastery as GRR [11–13]. Mastery measures a person’s perceived ability to cope that is the extent to which a person feels that he or she has control over his or her life chances and changes in behaviour or if these are controlled by fatalistic thoughts. Measures of mastery are associated with psychological and physical health. This has been demonstrated, especially for the incidence of myocardial infarction and in health behaviour [14, 15]. Additionally, low SES, in terms of economic difficulties, is related to lower mastery [16].

Vitality, the individual state of mind with feelings of energy and happiness, can also be seen as GRR [17]. The perception of vitality is important and beneficial in different ways, as it is related to physiological and psychological energy, happiness and life satisfaction. Moreover, it can increase a person’s coping ability and improve his or her performance in various areas. As such, vitality can be an indicator of health and well-being [18]. In the following discussion, we will refer to the perception of vitality as vitality.

PA is one of the main health-related behaviours, and regular PA is well known to improve health and reduce the risk of NCD and all-cause mortality [19, 20]. PA can be defined as all physical activities performed by skeletal muscles that result in increased energy consumption above basal metabolic rate [21]. Changes in levels of PA are related to overall changes in a person’s life, and positive experiences when performing PA can increase control over one’s PA [22]. High PA levels are also associated with better control over life chances and other behavioural changes, while low PA levels are associated with more fatalistic thoughts [23–25]. PA can also have a positive impact on energy levels and vitality, with a high level of PA in the general population being associated with high levels of energy and vitality [26].

In primary health care settings, health dialogues are one way to work with health promotion. A health dialogue is based on a person’s perspective of his or her situation and level of motivation to make health behaviour changes [27]. The health dialogue is an opportunity to support a higher level of motivation to increase levels of PA [28].

A previous study has demonstrated that high levels of PA in an adult population can compensate, to some extent, for a poor economic situation with regard to self-rated health (SRH) [29]. However, there is little knowledge about the characteristics of the psychological resources among those who, despite a less favourable socio-economic situation, are physically active. This study focuses on the psychological resources that are also considered as GRRs. More knowledge about these could be useful for health-promoting encounters and to increase levels of PA in sedentary groups, with the aim of improving their health.

### Aim

The aim of this study is to investigate the associations between PA level and mastery and vitality, respectively, within an adult population with self-reported economic difficulties.

## Materials and method

### Setting and data collection

The data in this cross-sectional study are baseline data from the Living Condition Stress and Health (LSH) study [29], which examines the importance of psychological factors on SES differences in health. The study population consisted of a randomly selected sample of people aged 40, 45, 50, 55, 60, 65 and 70 years. The participants, in south-eastern Sweden, were invited to their primary health care centre for a targeted health dialogue between 2012 and 2015, with the aim of decreasing the incidence and premature deaths from CVD. Those who participated in the targeted health dialogue were also invited to the LSH study. The targeted health dialogue was based on a special pedagogically designed graphical health tool illustrating the results from different questionnaires, anthropometric measures and blood tests. The standard process for the targeted health dialogue procedure has been described elsewhere [30–32]. An additional questionnaire with a set of instruments about psychological factors was provided, besides the ordinary health dialogue procedure, to the participants in the LSH study. Of the 28,702 persons invited to participate in a health dialogue and the LSH study, 12,164 (42%) accepted participation in a health dialogue, and of those 6860 (56%) persons (2980 men and 3880 women) chose to participate in the LSH study. The mean age of the participants was 54 years (SD of 9.9 years) [29].

### Analytical sample

The present study only included participants with self-reported economic difficulties. Self-reported economic difficulties were retrieved from one question on the questionnaires. The responses to the question *‘Is the economy a problem for you?’* were dichotomised into two categories. The answers *‘partly’* and *‘yes’* were combined into the category *‘Economic difficulties’* and the answer *‘no’* was defined as *‘No economic difficulties’.* Participants were excluded from the study if they responded in the questionnaire that they had been diagnosed (by a physician) with myocardial infarction or stroke, angina pectoris, chronic lung disease, rheumatoid arthritis, musculoskeletal disorders, neurological disease or depression, as these diseases could have had a negative influence on their capacity to be physically active. From the group with self-reported difficulties 480 were excluded due to one or more of these diseases. In total, the remaining analytical sample comprised 817 participants (482 women, 335 men).

### Outcome variables

In this study, mastery and vitality are used as outcome variables.

#### Mastery

The Pearlin Mastery Scale was used. Mastery comprises the ability to cope with life and the extent to which a person has control over his or her life chances or whether these are controlled by fatalistic thoughts [24]. A scale score for mastery has been constructed from six questions proposed by Pearlin [24]: To what extent are the following statements accurate for you? *‘There is really no way I can solve the problems I have’, ‘I have little control over things that happen to me’, ‘I often feel helpless in dealing with the problems of life’* and *‘There is little I can do to change many of the important things in my life’* with answers on a 4-point Likert-type scale ranging from *Not at all* (3 points) to *To a major extent* (0 points), and *‘I can do just about everything I set my mind to do’* and *‘What happens to me in the future mostly depends on me’* with answers on a 4-point Likert-type scale ranging from *Not at all* (0 points) to *To a major extent* (3 points).

One item on Pearlin’s original scale for mastery, which contains seven items, ‘*Sometimes I feel I’m being pushed around in life’,* was excluded from the present study, because it has been shown to be hard to understand and answer in Swedish society today. Excluding this item has been shown not to reduce the scale’s validity on group level [33, 34]. The reduced version of the Pearlin Mastery scale has a Cronbach’s alpha of 0.72 [34]. The Pearlin Mastery Scale has been proved valid in a healthy population [33]. Furthermore, the mastery questions were included in a specific questionnaire for participants in the LSH study.

In this study, all six questions had to be answered for the Mastery Scale score to be calculated. Before calculating them, all the scores for negative items were inverted. The Mastery Scale score runs from zero to eighteen, with a high score indicating control over life and a low score indicating more fatalistic thoughts on life.

#### Vitality

RAND-36 was included in the ordinary questionnaire for health dialogues. Scale scores for vitality were calculated using the four questions in the module for vitality (energy/fatigue) in RAND-36, following the instructions for the RAND-36 index [35]. The scale for vitality (energy/fatigue) in RAND-36 has a Cronbach’s alpha of 0.89 and has also shown good responsiveness [35]. The first two questions were *‘Did you feel full of pep?’* and *‘Did you have a lot of energy?*’ with the answers on a 6-point Likert-type scale ranging from *All of the time (100),* to *None of the time (0),* and the other two questions were *‘Did you feel tired?’ and ‘Did you feel worn out?’,* with the answers on a 6-point Likert-type scale ranging from *All of the time (0),)* to *None of the time (100).* All four questions must be answered to calculate the index for vitality. The scale range for vitality runs from 0 to 100 according to the RAND-36 index, using the average of the points from the four questions for vitality and fatigue, where a high score indicates high vitality.

### Independent variable

#### Physical activity (PA)

Information about PA was also derived from the questionnaires, with the amount of PA during leisure time and PA commuting to work being calculated and summarised. The person conducting the health dialogue also asked supplementary questions about PA and the intensity of performing PA, which means that the measurement of PA was a combination of answers in a questionnaire and an interview. The answers were transferred into ‘points’ of PA, representing the participants’ level of PA. Further details concerning the questionnaires and the calculation of PA scores can be found in Additional file 1 and have been described elsewhere [32]. The questionnaire has also been validated against Body Mass Index (BMI), Waist-Hip Ratio (WHR) and cholesterol, where higher points for PA were associated with lower BMI, WHR and cholesterol [36]. In this study, the PA points were divided into three groups: low PA (0–499 points), intermediate PA (500–999 points) and high PA (1000 points or more). One thousand points (high PA) corresponds to approximately 30 min of brisk walking per day, which is just over the World Health Organisation’s (WHO) recommended amount of at least 150 min of PA per week [20].

#### Confounding factors

As potential confounding variables, sex, age, smoking and food quality were considered, as it is well known that they are associated with SES and PA [37, 38]. Age was divided into seven groups, the same as for the recruitment groups: 40, 45, 50, 55, 60, 65 and 70 years old. For smoking habits, the question in the questionnaire had five response alternatives that were used in the analyses: *‘I have never smoked’, ‘I stopped smoking more than 6 months ago’, ‘I stopped smoking less than 6 months ago’, ‘I smoke but not daily’* and *‘I smoke daily’.* The question of food quality was captured from the questionnaire ‘20 questions about your food habits’, which estimated the intake of hard fats, fibre and sugar. Further details concerning this measure of food quality have been described elsewhere [32]. In this study, the food quality levels were divided into three groups, with 3–5 points meaning high food quality (characterised by a low intake of hard fats and a high intake of fibre), 6–8 points classed as intermediate food quality and 9–11 points highlighting low food quality (characterised by a high intake of hard fats and a low intake of fibre).

### Statistical analyses

Descriptive statistics were used to describe the background characteristics of the participants using proportions for categorical variables and mean levels, and standard deviations (SD) and scale scores for the two measures of resources of mastery and vitality for the three PA levels. Thereafter, linear regression analysis was used to describe associations between PA levels and scale scores for mastery and vitality, using the unstandardised regression coefficient (*β*) and 95% confidence intervals (95% CI). Unadjusted estimates (Model I), estimates adjusted for sex and age (Model II) and estimates adjusted for sex, age, smoking and food quality (Model III) were all derived. The regression analyses included participants with complete data on all variables. All the regression models used dummy variables for PA levels. The adjusted *R*^2^-value and the standardised Beta coefficients are provided for each model. Scatterplots of standardised residuals were used to check that the residuals roughly followed a normal distribution. A *p*-value of less than 0.05 was considered statistically significant. All the analyses were performed using SPSS version 26 (IBM Corp, Armonk, New York, USA).

## Results

### Characteristics of the study population

Table 1 shows the background characteristics of the 817 participants included in the study. The proportions of male participants in the groups with low, intermediate and high PA were 45.8, 40.1 and 39.6% respectively (Table 1). The mean age was 50.73 (SD 9.34) years. The proportions of daily smokers in these three groups with low, intermediate and high PA were 22.4, 8.2 and 7.4% respectively, and for food quality, the proportions of low food quality were 37, 29.8 and 29.1% respectively (Table 1).

**Table 1:** Background characteristics of included participants in a population with self-reported economic difficulties

Variable	Characteristics	Low PA *n* = 177 n (percent)	Intermediate PA *n* = 147 n (percent)	High PA *n* = 493 n (percent)
**Sex**	male	81 (45.8)	59 (40.1)	195 (39.6)
	female	96 (54.2)	88 (59.9)	298 (60.4)
**Age**	40 years	59 (33.3)	39 (26.5)	131 (26.6)
	45 years	22 (12.4)	13 (8.8)	60 (12.2)
	50 years	37 (20.9)	33 (22.4)	130 (26.4)
	55 years	19 (10.7)	13 (8.8)	35 (7.1)
	60 years	20 (11.3)	28 (19)	80 (16.2)
	65 years	12 (6.8)	8 (5.4)	22 (4.5)
	70 years	8 (4.5)	13 (8.8)	35 (7.1)
**Smoking habits**	Never smoked	85 (48.9)	80 (54.8)	272 (56.2)
	Stopped > 6 months ago	43 (24.7)	46 (31.5)	146 (30.2)
	Stopped < 6 months ago	3 (1.7)	3 (2.1)	4 (0.8)
	Smokes but not daily	4 (2.8)	5 (3.4)	26 (5.4)
	Daily smoker	39 (22.4)	12 (8.2)	36 (7.4)
**Food quality**	High food quality	52 (31.5)	53 (40.3)	161 (35.5)
	Intermediate food quality	52 (31.5)	39 (29.8)	161 (35.5)
	Low food quality	61 (37.0)	39 (29.8)	132 (29.1)

Data from the LSH study Sweden

The mean values for mastery and vitality for the different PA levels are shown in Table 2.

**Table 2:** Mastery and vitality in the different physical activity (PA) levels in people reporting economic difficulties

	Low PA mean (SD)	Intermediate PA mean (SD)	High PA mean (SD)
Mastery *n* = 488	11.34 (2.88)	12.08 (2.59)	12.04 (2.74)
Vitality *n* = 757	48.09 (23.10)	55.04 (21.73)	56.72 (21.92)

Data from the LSH Study Sweden

The associations between PA levels and mastery and vitality respectively were further analysed with linear regression analyses in both unadjusted and adjusted models, as shown in Table 3. A high level of PA was associated with higher scores for mastery, also after adjustment for sex, age, smoking and food quality, (*β* = 0.72 [95% CI 0.08 to 1.37]) compared with low PA. For intermediate PA, the estimated beta coefficient pointed towards an association in the same direction. However, this association was not statistically significant (Table 3).

**Table 3:** Association between physical activity (PA) levels and scale scores of mastery

	Model I *n* = 446 *R*^2^ = 0.004	*P* value	Standardised Beta coefficient
	*β*-coefficient and 95% CI		
**Low PA (ref)**	1		
**Intermediate PA**	0.56 (− 0.26 to 1.38)	0.182	0.078
**High PA**	0.64 (−0.004 to 1.28)	0.051	0.11
	**Model II**		
	*n* = 446 *R*^2^ = 0.008		
	*β*-coefficient and 95% CI	*P* value	Standardised Beta coefficient
**Low PA (ref)**	1		
**Intermediate PA**	0.61 (−0.21 to 1.43)	0.145	0.09
**High PA**	0.67 (0.03 to 1.31)	0.040*	0.12
**Sex**	−0.04 (− 0.56 to 0.48)	0,876	− 0.07
**Age**	−0.03 (− 0.05 to − 0.00)	0.053	−0.09
	**Model III**		
	*n* = 446		
	*R*^2^ = 0.025		
	*β*-coefficient and 95% CI	*P* value	Standardised Beta coefficient
**Low PA (ref)**	1		
**Intermediate PA**	0.62 (−0.20 to 1.44)	0.135	0.09
**High PA**	0.72 (0.08 to 1.37)	0.028*	0.13
**Sex**	−0.08 (− 0.60 to 0.43)	0.753	− 0.02
**Age**	−0.03 (− 0.06 to 0.00)	0.029*	−0.10
**Smoking habits**	−0.20 (− 0.42 to 0.01)	0.064	0.09
**Food Quality**	0.41 (0.10 to 0.72)	0.010*	0.12

The LSH study, Sweden. The association between physical activity levels and scale scores of mastery in a population with self-reported economic difficulties, estimated by linear regression analysis using low physical activity levels as reference level (ref). Unstandardised regression *β*-coefficients and 95% confidence intervals (CI), adjusted *R*^2^-values and standardised Beta coefficients are presented. Model I=Unadjusted analysis; Model II = Adjusted for sex and age; Model III = Adjusted for sex, age, smoking habits and food quality

Higher levels of PA were also associated with higher scores for vitality. In the adjusted model (Model III), the beta coefficient was 6.70 [95% CI 1.40 to 12.0] for intermediate PA and 9.30 [95% CI 5.20 to 13.40] for high PA (Table 4).

**Table 4:** Association between physical activity (PA) levels and scale scores of vitality

	Model I *n* = 683 *R*^2^ = 0.026	*P* value	Standardised Beta coefficient
	*β*-coefficient and 95% CI		
**Low PA (ref)**	1		
**Intermediate PA**	7.14 (1.80 to 12.48)	0.009*	0.12
**High PA **	9.42 (5.30 to 13.53)	< 0.001*	0.21
	**Model II**		
	*n* = 683 *R*^2^ = 0.067		
	*β*-coefficient and 95% CI	*P* value	Standardised Beta coefficient
**Low PA (ref)**	1		
**Intermediate PA**	6.79 (1.55 to 12.04)	0.011*	0.12
**High PA**	9.34 (5.3 to 13.38)	< 0.001*	0.21
**Sex**	−5.49 (−8.77 to −2.21)	0,001*	−0.12
**Age**	0.39 (0.22 to 0.57)	< 0.001*	− 0.17
	**Model III**		
	*n* = 683		
	*R*^2^ = 0.065		
	*β*-coefficient and 95% CI	*P* value	Standardised Beta coefficient
**Low PA (ref)**	1		
**Intermediate PA**	6.70 (1.40 to 12.00)	0.013*	0.11
**High PA**	9.30 (5.20 to 13.40)	< 0.001*	0.20
**Sex**	−5.54 (−8.83 to −2.26)	0.001*	−0.12
**Age**	0.39 (0.21 to −0.56)	< 0.001*	0.16
**Smoking habits**	−0.46 (−1.33 to 1.23)	0.94	0.00
**Food Quality**	1.01 (0.99 to 3.01)	0.32	0.04

The LSH study, Sweden. The association between physical activity levels and scale scores of vitality in a population with self-reported economic difficulties, estimated by linear regression analysis using low physical activity as reference level (ref). Unstandardised regression *β*-coefficients and 95% confidence intervals (CI), adjusted *R*^2^-values and standardised Beta coefficients are presented. Model I=Unadjusted analysis; Model II = Adjusted for sex and age; Model III = Adjusted for sex, age, smoking habits and food quality

## Discussion

In this study, based on an adult population with self-reported economic difficulties, we observed that higher levels of PA were associated with higher scale scores of mastery and vitality. It is well known that people with low SES are often less physically active compared with people in higher socio-economic groups [39, 40]. However, there is limited knowledge about which psychological resources characterise physically active people with low SES.

This study contributes information that we believe is important to health promotion work with targeted health dialogues aimed at improving PA and health within low SES groups. However, it should be noted that due to the cross-sectional design of this study, we cannot establish the direction of the association between PA versus mastery and vitality and hence are prevented from drawing any conclusions about causality.

In this study, high level of PA, was associated with higher level of mastery and vitality among people with economic difficulties. As pointed out above, we could not establish the direction of the association due to the cross-sectional design.. It is known from other studies of general populations that people who are physically active report higher scale scores of mastery in terms of control over life chances and behaviour change and have fewer fatalistic thoughts. They also report higher scores for vitality, which is most probably a result of the beneficial psychological and physiological effects of being physically active [41–44]. Still, the availability of psychological resources, in terms of high mastery and high vitality, may also be important to improving motivation and the capacity to become more physically active. Additionally, vitality in terms of perceived benefits from being physically active is important for the attitude to and likelihood of performing PA [39, 45–47].

In the present study, it was observed that high PA, as described reaching the WHO recommendations for PA or over, is associated with higher degree of mastery, among people reporting economic difficulties. One of the main aims of targeted health dialogues is to support individuals to increase their PA. One essential and important part is the way this targeted health dialogue is conducted. Our results suggest that the method of motivational interviewing must encompass salutogenic strategies to support their behavioural changes towards increasing PA, as well as to support people with weak psychological resources to increase mastery or internal locus of control (LOC). Earlier studies in a general population aged 15 to 69 years from all SES groups have found that people with a higher level of PA also have higher levels of mastery or internal LOC [48]. Antonovsky linked mastery to internal LOC and internal LOC to a resource from the salutogenic perspective [12]. Pearlin’s mastery questions, the measure used in this study, have also previously been used to investigate LOC [45]. Moreover, earlier studies have demonstrated that it is possible to increase mastery over the course of life. Intervention programmes aimed at strengthening perceived control over life and thoughts of what individuals can do to improve things in life have been successful and shown to improve health behaviour [15].

Our study also showed clear associations between PA and vitality. Vitality is a subjective feeling of both mental and physical energy and an indicator of health [18, 47]. A higher perceived vitality is a benefit that comes after PA and could be a reason to continue to or increase PA. Even a small amount of PA like a brisk walk for 10 min could increase vitality for 2 h after completion of the activity [44]. Earlier studies with the same instrument for vitality as used in our study showed that a high level of PA reduced the risk of low vitality by 41% compared with a sedentary lifestyle [49]. Thus, PA is a health-related behaviour that affects both physical and mental well-being and hence plays a part in health promotion to ‘go beyond healthy life-styles to wellbeing’ [20].

## Strengths and limitations

The study sample is part of the population-based study LSH, with many participants randomly drawn from the general population who are representative of an adult population of both men and women. Therefore, the studied population, which comprises people reporting economic difficulties, is most probably representative of people in Sweden who perceive that they have economic difficulties. There are studies on the association between physical activity and mastery as well as vitality in general populations, but to the best of our knowledge, there are no such studies in a population with self-reported economic difficulties.

As both PA and vitality can be affected by somatic problems and diseases [50] we excluded participants who had reported diseases that could have an impact not only on PA but also on vitality. All data in this study were based on self-reports, which could be regarded as a limitation. Both the psychological resources and PA were measured with established instruments [32, 33, 35]. In addition to the PA questionnaire, the health care professionals conducting the health dialogue with the participants asked supplementary questions about PA in an attempt to obtain more detailed information about PA. This approach may obtain more valid answers regarding PA compared with using only the questionnaire.

About 60% of the participants in this study reported a high PA level (1000 points or over). As mentioned in the method part, 1000 points (high PA) corresponds to just over the WHO recommended amount of at least 150 min of PA per week [20]. In a Swedish context, this is comparable to the results from the national health survey, in which one-third of the population did not achieve 150 min per week of PA [51]. In the subpopulation with economic difficulties, examined in this study, the proportion of participants being physically active can be considered as fairly high. However, since we excluded people with a present disease, a slightly higher proportion being physically active could be expected in the remaining sample. An earlier study based on the LSH study showed differences in PA between participants with and without economic problems, where participants without economic problems took a higher amount of PA [29].

The primary aim of this study was to investigate the association between PA and mastery and vitality, respectively, after taking into account several potential confounding variables that may influence the association. Thus the aim was not to find a model that best predicts the outcome variables. Obviously, there are more variables than PA that are associated with and help to explain the variance of mastery and vitality, which is also seen in the rather low *R*^2^-values in the analyses. It should also be noted that the covariates in the Model II and Model III did not affect the association between PA and mastery and vitality, respectively, to any major extent.

The response rate for the mastery questions was lower than for vitality. This is because the Mastery Scale, together with other instruments on psychological factors and working life but not RAND, was included in a separate questionnaire for which the response rate was lower. Furthermore, one question was not relevant and was removed from the scale due to the fact that it was not relevant in a Swedish context, and that might have influenced the interpretation of the original scale for individuals, even if the validity on group level was not reduced [34]. However, in this context, it could strengthen the coherence of the scale [33].

Finally, as discussed above, this study is cross-sectional, and to further understand the investigated associations, studies with longitudinal design or intervention studies are needed.

## Conclusions

We found in an adult population with self-reported economic difficulties that higher levels of physical activity were related to higher scale scores of both mastery and vitality. Our results support the importance of tools and methods aimed at strengthening and promote physical activity and psychological resources such as mastery and vitality in interventions, for instance in the context of targeted health dialogues. This is even more important among people with low SES in terms of economic difficulties.

## Supplementary Information

**Additional file 1.**

## Data Availability

The datasets generated and/or analysed during the current study are not publicly available due to privacy and ethical restriction, but are available from the corresponding author on reasonable request.

## Abbreviations

*β*: Regression coefficient
CI: Confidence intervals
GRR: General resistant resources
LOC: Locus of control
NCD: Non-communicable diseases
PA: Physical activity
Ref: Reference value
SES: Socio-economic-status
SRH: Self-rated Health
SD: Standard Deviations
WHO: World Health Organisation
